# miR-30a-5p inhibits osteogenesis and promotes periodontitis by targeting Runx2

**DOI:** 10.1186/s12903-021-01882-9

**Published:** 2021-10-11

**Authors:** Xiangdong Liu, Bo Yang, Yan Zhang, Xiaorui Guo, Qianjuan Yang, Xiaojing Liu, Qingxia Bai, Qun Lu

**Affiliations:** 1 State Key Laboratory of Military Stomatology and National Clinical Research Center for Oral Diseases and Shaanxi Engineering Research Center for Dental Materials and Advanced Manufacture, Department of Implant Dentistry, School of Stomatology, The Fourth Military Medical University, Xi’an, 710032 China; 2 State Key Laboratory of Military Stomatology and National Clinical Research Center for Oral Diseases and Shaanxi Key Laboratory of Oral Diseases, Department of Operative Dentistry and Endodontics, School of Stomatology, The Fourth Military Medical University, Xi’an, China; 3 Shanxi Provincial People’s Hospital, Shanxi, China; 4 Baoding First Central Hospital, Baoding, China; 5 The Fourth Affiliated Hospital of Harbin Medical University, Harbin, China

**Keywords:** Periodontitis, miRNA, Runx2, Rescue

## Abstract

**Background:**

Periodontitis is the most extensive chronic inflammatory bone resorption disease. MiRNAs offer a potential way for potential therapy. Indeed, miR-30a-5p had an increasing expression in periodontitis gingivae, but whether it promotes osteogenesis and inhibits inflammation remains unknown.

**Methods:**

Periodontitis model was exhibited by wire ligation and verified by micro-CT and HE staining; qPCR was used to detect the expression of miR-30a-5p; miR-30a-5p inhibitors and mimics were transfected into MC3T3-E1 cell line by lipofectamine 3000; The dual luciferase reporter gene experiment and RIP experiment were used to detect the relationship between miR-30a-5p and Runx2; Rescue experiment was used to verify the relationship between miR-30a-5p and Runx2.

**Results:**

Periodontitis model was exhibited successfully and miR-30a-5p was overexpressed at the bone and gingival tissues of this model. miR-30a-5p inhibitors not only promoted the osteogenesis but also relieved inflammation. Runx2 is a target of miR-30a-5p by dual luciferase reporter gene experiment and RIP experiment. Rescue experiments revealed that miR-30a-5p inhibitors would promote osteogenesis and relieve inflammation by targeting Runx2 in inflammation of MC3T3-E1 cell line.

**Conclusions:**

That all suggested that miR-30a-5p-mediated-Runx2 provided a novel understanding of mechanism of periodontitis.

**Supplementary Information:**

The online version contains supplementary material available at 10.1186/s12903-021-01882-9.

## Background

Periodontitis, which accompanied with pathologic loss of soft and hard tissue [1], is related to the mutual effects between host immunity and dysfunctional oral microbiota located in the subgingival niche [2–4]. In developing countries, periodontitis is more prevalent than developed countries [5]. Uncontrolled periodontitis may cause teeth loss, diabetes mellitus [6], preterm low birthweight [7] and cardiovascular disease [8], so it is important to intervene it early [9]. MicroRNA (miRNA) is a non-coding RNA and plays the role of silencing the target genes through complementary base pairing [10, 11]. Several serum miRNAs including miR-207, miR-495, miR-376b-3p may be the biomarkers of periodontitis [12]. A research by miRNA microarray showed that miR-30a-5p increased in periodontitis [13]. Furthermore, there are also many different miRNAs involved in the process and pathology of periodontitis [14, 15]. MiR-21 has been reported to inhibit the inflammation and down-regulate in periodontitis [16]. miR-155-5p/SIRT1 regulatory network alleviated inflammatory of PDLSCs [17]. MiR-30a-5p, which located in chromosome 6:71, involved aspects of diseases and physiology. miR-30a-5p combined miR-769-59 could be a biomarker from plasma for diagnosis in in oral squamous cell carcinoma (OSCC) [18]. But whether miR-30a-5p influenced the biological functions of periodontitis remains unknown.

Runx2 (the osteoblast transcription factor 2) is a transcription factor for osteoblast differentiation [19]. Komori et al. [20] found that osteoblasts were almost absent in Runx2^−/−^ mice. Meanwhile, it plays a vital role in chondrocyte maturation [21]. For special deletion of Runx2^−/−^of skeleton, chondrocyte maturation was severely inhibited [22]. Overexpression of Runx2 accelerated progression of post-traumatic osteoarthritis in adult mice [23]. Runx2 improved osteogenesis of bone marrow mesenchymal stem cells (BMSCs) in vitro and repaired cartilage repair in vivo [24]. A recent study indicated that rs59983488 in Runx2 was associated with persistent apical periodontitis (PAP) for a higher risk of developing PAP [25]. However, whether miR-30a-5p targeting Runx2 influences the process of periodontitis remains unknown.

In this study, we designed the animal model of periodontitis and aimed to investigate the expression of miR-30a-5p and its effects on the osteogenesis and inflammation by targeting Runx2 in periodontitis.

## Methods

### Ethics statement

All experiments performed in this study were confirmed by the Ethical Committee of the School of Stomatology, The Fourth Military Medical University (Xi’an, China, NO: kq-013) and in compliance with ARRIVE guidelines (https://arriveguidelines.org). All methods were accordance with relevant guidelines including the revised Animals (Scientific Procedures) Act 1986 in the UK and Directive 2010/63/EU in Europe. All lab work and methods were performed by the authors of this paper.

### Animal model

32 SD male rats (250 g ± 10 g) in the Experimental Animal Center of the School of Stomatology, The Fourth Military Medical University were selected and adaptive fed in a SPF (Specific Pathogen Free) condition with temperature of 21–23 °, relative humidity of 30–60% and 12 h lighting/dark alternating environment.

Periodontitis models built by the ligature-induced method, which has been widely used in the previous studies [4]. Firstly, the rats were anesthetized by intraperitoneal injecting 5 mg/kg xylazine (Albrechts GmbH, Aulendorf, Germany) and 85 mg/kg ketamine (Albrechts GmbH, Aulendorf, Germany) into the abdominal cavity to anesthetize them, then the periodontal tissue of the maxillary 1st molar were separated and rooted with 0.2 mm orthodontic ligation wire, periodontal tissue on the right was used as control group. Rats were sacrificed by overdose anesthesia and organs were collected for pathological examination.

### Micro-computed tomography (micro-CT)

Maxillary bone specimens were dissected at 0, 7 days and 21 days after ligation and fixed into 4% paraformaldehyde, respectively. Samples were analyzed of microstructures of bone loss by the micro-CT system (Siemens, Inveon, Germany) in the maxillary area, which were scanned at parameters of 80 kV and 80 μA with a pixel size of 18 μm and an exposure time of 3000 ms. GEHC Micro View software was used to reconstruct the 3D images after scanning.

### HE and TRAP staining

After micro-CT scanning, the specimens were decalcified in 17% EDTA solution (Thermo scientific, Waltham, MA USA) for 30–40 days. After dehydration, paraffin embedding was performed, and 5 μm continuous sections were made and nearly 5 μm continuous sections were made. HE and TRAP staining were performed on the sections at three locations according to the manufacturer’s protocols.

### Cell culture and LPS inducing

MC3T3-E1 and 293 T cell lines were obtained from ATCC (ATCC, Manassas, VA, USA). All cell lines were cultured in RPMI-1640 (Sigma) with 10% FBS (Sigma), 1% 100 units/ml penicillin and 100 μg/ml streptomycin (Sigma) in an incubator (37 °C, 5% CO_2_) (NUAIRE, Plymouth, MN, USA). For simulation of periodontitis in vivo, 1ug/ml lipopolysaccharide (LPS) from Porphyromonas gingivalis were added at serum-free medium of MC3T3-E1 cell line.

### MiRNA-30a-5p mimic/inhibitor and si-Runx2 transfection

MC3T3-E1 cell line was plated into 6-wells plate after transfected with 1.2 × 10^5^ cells/cm^3^. After 24 h, cells were washed by 1 × PBS (Sigma) three times and configured with transfection solution (10ul opti-Mem medium + 0.2ul lipofectamine 3000 (Promega, Madison, Wisconsin, USA) was prepared with liquid A; 10 ul Medium + 100 nM MiR-30a-5p mimics, inhibitor, si-Runx2/NC solution were liquid B) (Genepharm, Suzhou, China). miR-30a-5p mimics, inhibitor and si-Runx2 were designed from Sangon Biotech company (Sangon Biotech, Shanghai, China). The sequences have been shown in Table 1. After preparation, Solution A and Solution B were mixed for 15 min at 1:1 ratio by volume at a room temperature, and the liposome complex was added to the cellular pore plate, supplemented with serum-free medium, and then incubated them in an incubator for 24 h.

**Table 1:** Sequencing of primers, mimics, inhibitors and siRunx2

Name	Forward	Reverse
miR-30a-5p	UGUAAACAUCCUCGACUGGAAG	CAGTGCGTGTCGTGGAGT
IL1-R1	CTGCTGTCGCTGGAGATTGAC	TTGGCAGGTACAAACCAAAGAT
IL1-R2	GTTTCTGCTTTCACCACTCCA	GAGTCCAATTTACTCCAGGTCAG
TNFR1	GGGGATACATCCATCAGGGGT	GCTCGGACAGTCACTCACC
ALP	CAGCGGGTAGGAAGCAGTTTC	CCCTGCACCTCATCCCTGA
Runx2	GACTGTGGTTACCGTCATGGC	ACTTGGTTTTTCATAACAGCGGA
OC	CCCATCTTTGAGCATCTTGGT	GCCCAGCCTGAGTAGTGAAG
CoL1	AATGGAAGTTCTACTCGCGTAGG	TTCTCGCCTGGTTGACCTTTG
GAPDH	AATGGATTTGGACGCATTGGT	TTTGCACTGGTACGTGTTGAT
miR-30a-5p mimic	UGUAAACAUCCUCGACUGGAAG	UCCAGUCGAGGAUGUUUACAUU
mimic NC	UUCUCCGAACGUGUCACGUTT	ACGUGACACGUUCGGAGAATT
Inhibitor NC	UUCUCCGAACGUGUCACGUTT	ACGUGACACGUUCGGAGAATT
Si-Runx2-492	CCGGGAAUGAUGAGAACUATT	UAGUUCUCAUCAUUCCCGGTT
Si-Runx2-909	GGUCCUAUGACCAGUCUUATT	UAAGACUGGUCAUAGGACCTT
Si-Runx2-1059	CACGCUAUUAAAUCCAAAUTT	AUUUGGAUUUAAUAGCGUGTT
U6	ATGGGTCGAAGTCGTAGCC	TTCTCGGCGTCTTCTTTCTCG

### Osteogenesis differentiation induction

Cells were cultured into 6-wells plate for 24 h and then were transfected miR-30a-5p mimics, inhibitor, si-Runx2, or si-Runx2 plus miR-30a-5p inhibitor and negative controls. Osteogenic induction solution (10 mmol/L sodium glycerophosphate, 50 g/mL ascorbic acid, 10 mol/L dexamethasone and 100 ml/L fetal bovine medium) was replaced 4 h and cells were cultured in a CO_2_ incubator.

### Alkaline phosphatase and Alizarin red staining

After osteogenic induction for 7 or 21 days, cell line was fixed with 4% paraformaldehyde (Sigma) for 30 min at a room temperature, and then washed 3–5 times with 1 × PBS for 3–5 min; Secondly, BCIP/NBT staining solution (Cyagen, Santa Clara, CA) or Alizarin red (Solarbio) were added to the cells and incubated at a room temperature for 5–30 min in the dark. Until the expected color appeared, BCIP/NBT or Alizarin red dye solution (Cyagen) were removed and washed 2–3 times with distilled water and taken pictures.

### Dual-luciferase reporter gene

To verify the targeting relationship between miR-30a-5p and Runx2, we constructed a dual-luciferase reporter vector (WT) and a mutant dual-luciferase reporter vector (MUT), respectively. Sequencing results were shown in the Additional file 1: Figure S1A-1B. miR-30a-5p mimic, WT and MUT were co-transfected into 293T cell line for 48 h. The luciferase activity measured using a Dual-Glo Luciferase Assay System and assayed with a Multimode Plate Reader with a normalization of Renin luciferase activity.

### RNA extraction and quantitative real-time polymerase chain reaction (qRT-PCR)

RNA from the cell line extracted from Trizol reagent according to the manufacturer’s protocols. A Nano-Drop spectrophotometer was used to measure the concentration of RNA; mRNA reversed into cDNA by Takara Prime Script RT reagent Kit with gDNA Remover, and miRNA reversed by using All-in-One miRNA qRT-PCR System according to the manufacturer’s protocols using the comparative CT (ΔΔCt) method. The primer information was shown in Table 1.

### RNA immunoprecipitation (RIP)

RIP was used to detect endogenous interaction between miR-30a-5p and Runx2. Firstly, cells were transfected into miR-30a-5p mimics and negative control, respectively. Cell lysates and magnetic beads were together incubated with anti-Argonaute2 (antibodies-online, Aachen, Germany) and eluted absolutely. The precipitated RNA was analyzed by qRT-PCR with an input as positive control and IgG as a negative control (Millipore, Darmstadt, Germany).

### Statistical analysis

Mean ± Standard Error of Mean (mean ± SEM) methods were used for all data, Student’s *t* test was carried out for statistical analysis using GraphPad Prism 9 (SPSS v.19.0, IBM, Chicago, IL, USA). Values of *P* < 0.05 were considered statistically significant.

## Results

### miR-30a-5p was up-regulated in gingiva tissues and bones in periodontitis of rat model

**Fig. 1:**
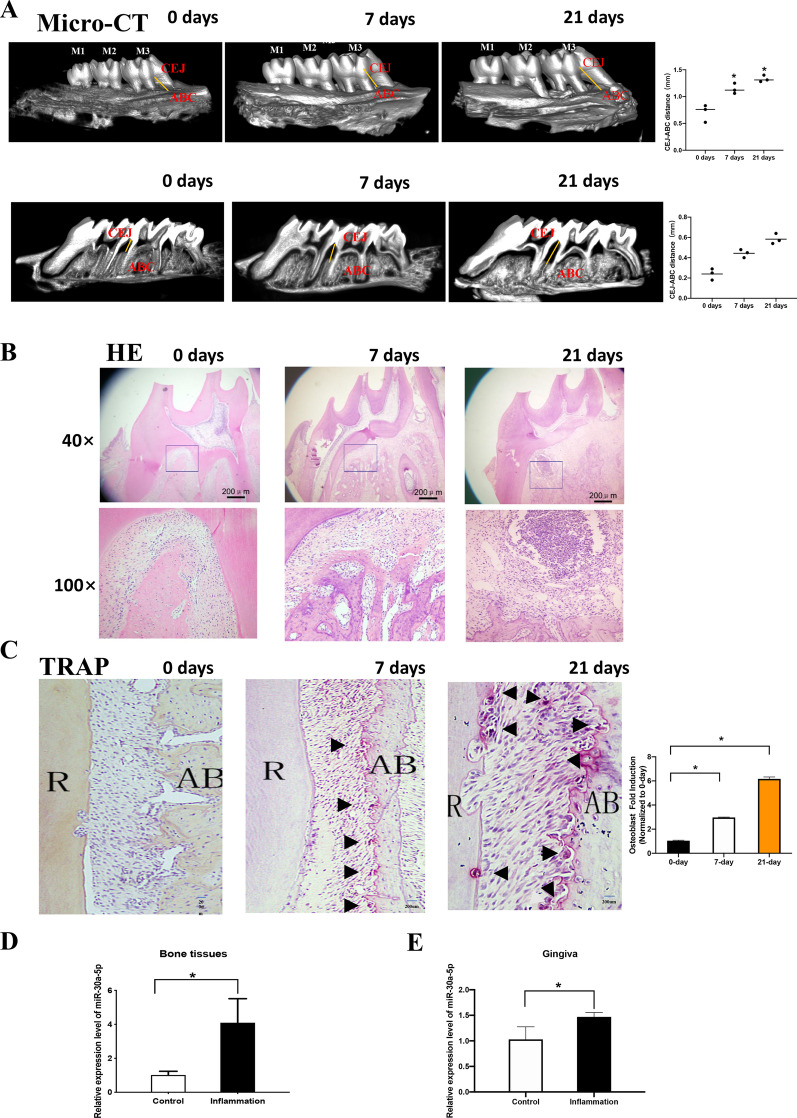
miR-30a-5p was upregulated at bone and gingiva in periodontitis rat model. **A** Bone loss was examined by micro-CT after ligation for 7, 21 days. **B** Cellular morphology was examined by HE after ligation for 0, 7, 21 days. **C** Osteoclasts were examined by TRAP after ligation for 0, 7, 21 days. **D** The expression of miR-30a-5p was examined by qPCR of bone tissues from periodontitis (inflammation) and healthy controls (control). **E** The expression of miR-30a-5p was examined by qPCR of gingival tissues from periodontitis (inflammation) and healthy controls (control). (R: root; AB: alveolar bone). All data are shown as mean ± SEM. *p < 0.05; **p < 0.01

For detecting the expression of miR-30a-5p, a rat model of periodontitis was exhibited by ligature-inducing. As micro-CT results showed in Fig. 1A, bone loss occurred after ligation-inducing for 7 days and a deeper loss in 21 days. HE and TRAP results indicated that inflammation cytokines and osteoclast cells appeared after ligation at 7 days and reached a higher expression level at 21 days (Fig. 1B, C). miR-30a-5p was upregulated in bone and gingiva tissues comparing with normal ones (Fig. 1D, E). It suggested that miR-30a-5p involved in the occurrence and development of periodontitis.

### miR-30a-5p was upregulated in MC3T3-E1 cell line after LPS inducing

**Fig. 2:**
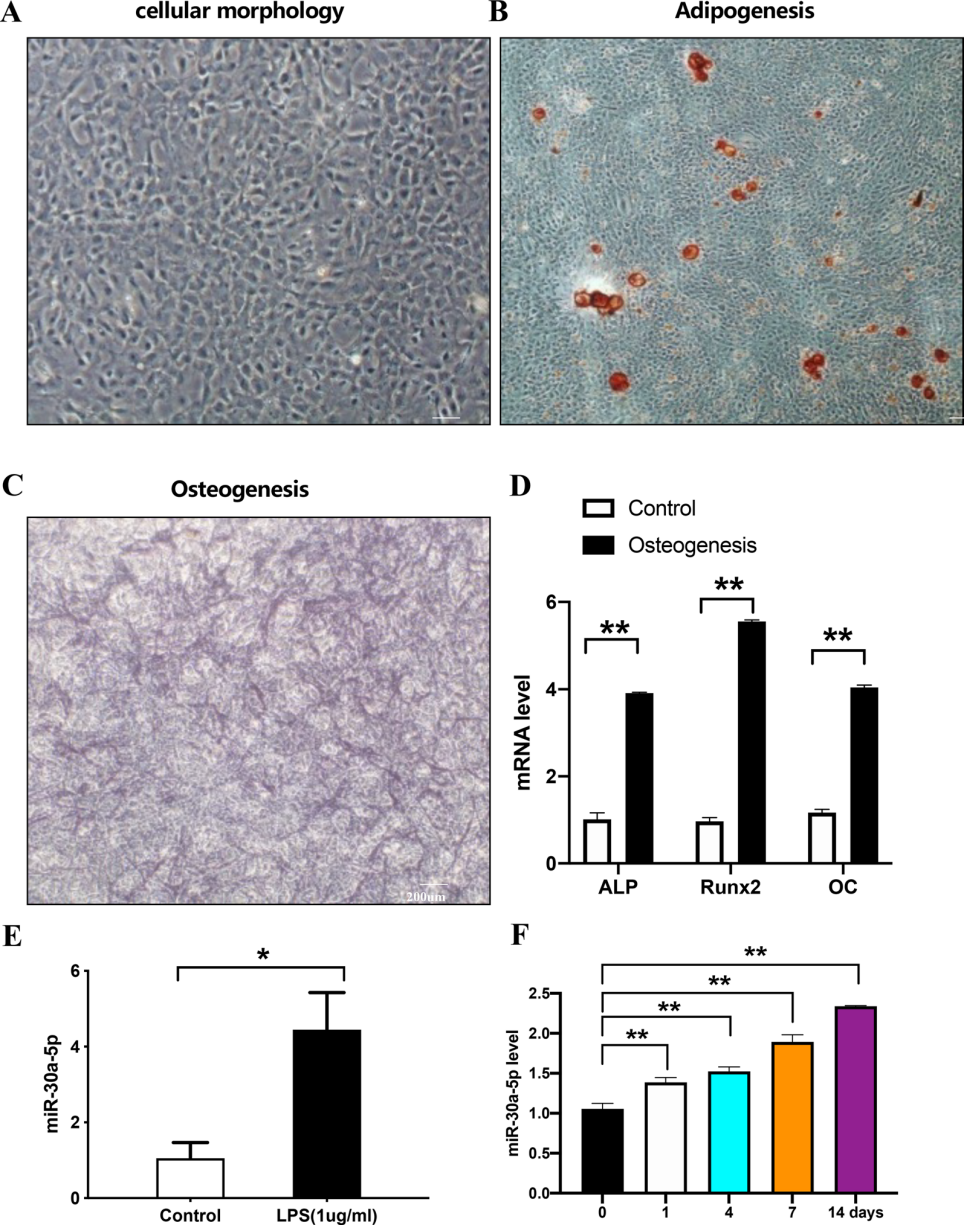
miR-30a-5p was upregulated in MC3TC-E1 cell line under LPS-inducing. **A** Microscopic image of P3 of MC3T3-E1 taken by microscope. **B** The level of lipid droplet was examined by oil-red staining in MC3T3-E1 cells after 14 days after adipogenic induing. **C** The level of ALP was examined by ALP staining in MC3T3-E1 cells after 14 days after osteogenic induing. **D** The expression of ALP, Runx2 and OC were examined by qPCR in MC3T3-E1 cells during osteogenic induing. **E** The expression of miR-30a-5p was examined by qPCR in MC3T3-E1 cells after LPS inducing (1ug/ml). **F** The expression of miR-30a-5p was examined by qPCR in MC3T3-E1 cells after LPS induing (1 ug/ml) for 0, 1, 4, 7, 14 days. All data are shown as mean ± SEM. *p < 0.05; **p < 0.01

MC3T3-E1 cell line were cultured (Fig. 2A) and oil red staining result showed its adipogenic tendency (Fig. 2B). Results of alkaline phosphatase (ALP) staining (Fig. 2C) and qPCR of osteogenic genes (Fig. 2D) showed its osteogenic differentiation. qRCR results showed that miR-30a-5p was upregulated in MC3T3-E1 cell line after LPS-inducing treatment (Fig. 2E). A much more increasing level could be seen in 1, 4, 7, 14 days with LPS-inducing (Fig. 2F).

### miR-30a-5p inhibitor reduced the expression of inflammatory factors and promoted the expression of osteogenesis factors

**Fig. 3:**
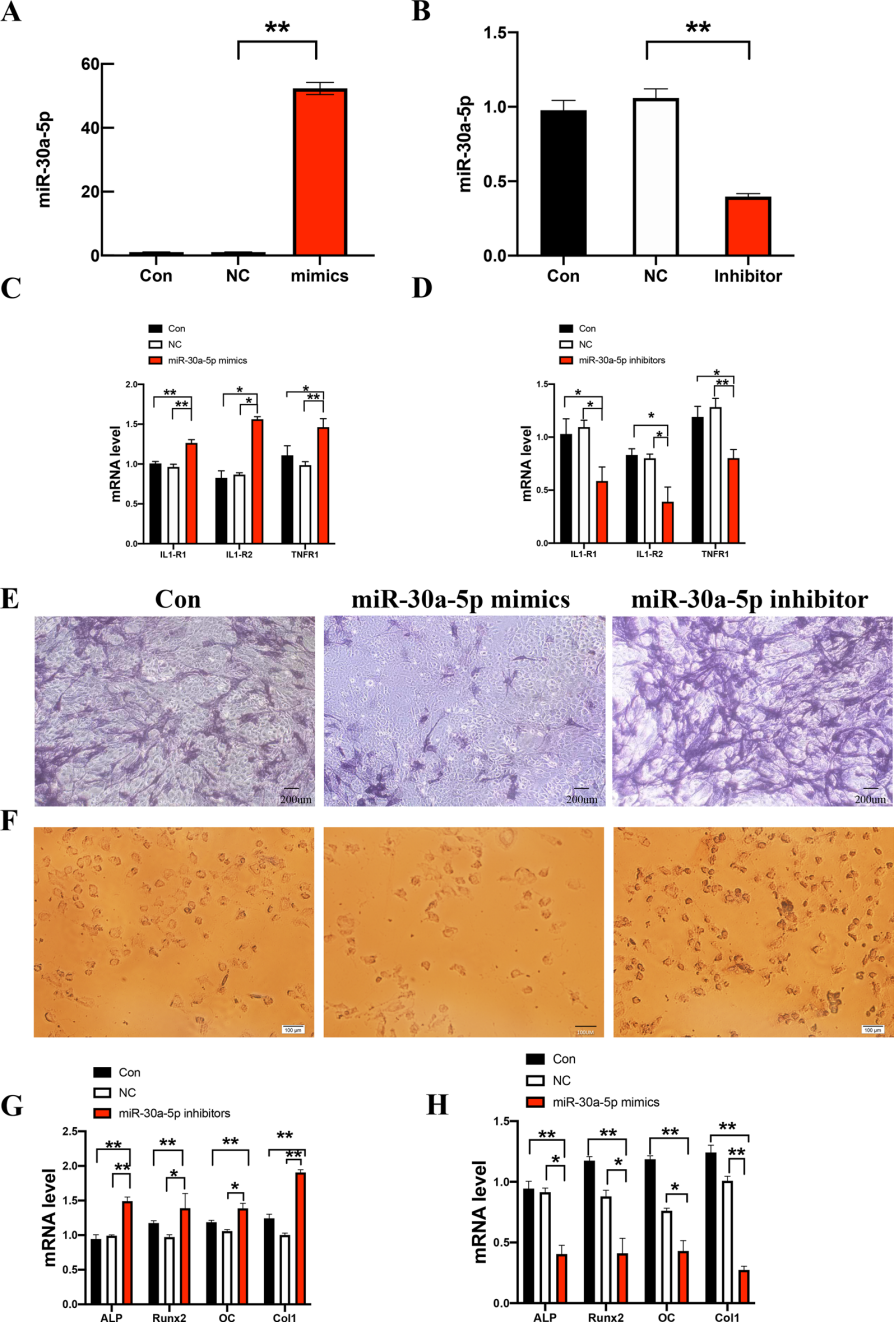
miR-30a-5p induced inflammation and inhibited osteogenesis. **A** The expression of miR-30a-5p was examined by qPCR in MC3T3-E1 cells after 24 h after transfected with relative controls (NC), miR-30a-5p mimics (mimic) and control. **B** The expression of miR-30a-5p was examined by qPCR in MC3T3-E1 cells after 24 h after transfected with relative controls (NC), miR-30a-5p inhibitors (inhibitors) and control. **C** The expression of IL1-R1, IL1-R2 and TNFR1 were examined by qPCR in MC3T3-E1 cells after 24 h after transfected with relative controls (NC), miR-30a-5p mimics (mimic) and control. **D** The expression of IL1-R1, IL1-R2 and TNFR1 were examined by qPCR in MC3T3-E1 cells after 24 h after transfected with relative controls (NC), miR-30a-5p inhibitor and control. **E** The level of ALP was examined by ALP staining in MC3T3-E1 cells after 7 days after transfected with miR-30a-5p mimics, miR-30a-5p inhibitors and relative controls (control). **F** The level of Alizarin red was examined by ALP staining in MC3T3-E1 cells after 21 days after transfected with miR-30a-5p mimics, miR-30a-5p inhibitors and relative controls (control). **G** The expression of ALP, Runx2, OC and Col1 were examined by qPCR in MC3T3-E1 cells after 24 h after transfected with relative controls (NC), miR-30a-5p inhibitor and control. **H** The expression of ALP, Runx2, OC and Col1 were examined by qPCR in MC3T3-E1 cells after 24 h after transfected with relative controls (NC), miR-30a-5p mimics and control. All data are shown as mean ± SEM. *p < 0.05; **p < 0.01

As showed in Fig. 3A, B, miR-30a-5p was upregulated in the mRNA level when transfecting miR-30a-5p mimics while downregulated in miR-30a-5p inhibitor comparing to relative control groups. The inflammatory factors IL1-R1, IL1-R2 and TNFR1 were upregulated when transfecting miR-30a-5p mimics (Fig. 3C), accompanied by downregulated transfecting miR-30a-5p inhibitors (Fig. 3D). MiR-30a-5p inhibitors promoted osteogenesis match along with miR-30a-5p mimics inhibited osteogenesis, based on results of Alkaline phosphatase and alizarin red staining (Fig. 3E, F). Osteogenic factors ALP, Runx2, OC, and Col-1 were upregulated when transfected miR-30a-5p inhibitor (Fig. 3G), accompanying by downregulated when transfected miR-30a-5p mimics (Fig. 3H), based on the result of qRT-PCR. These indicated that miR-30a-5p involved in regulation of osteogenesis and inflammation in periodontitis, and miR-30a-5p inhibitors relieved inflammation and promoted the osteogenesis.

### Runx2 is a target of miR-30a-5p

**Fig. 4:**
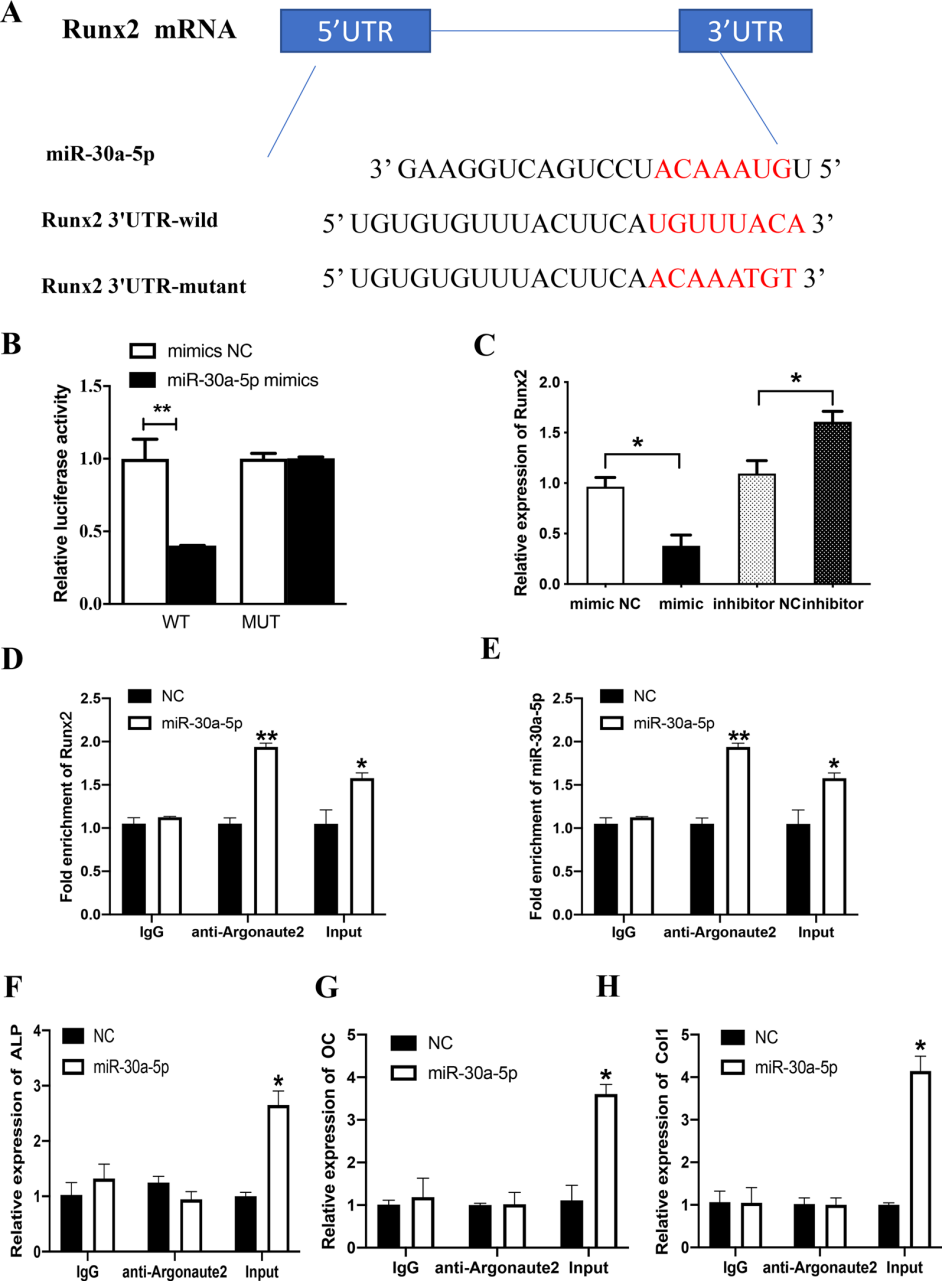
miR-30a-5p directly targets Runx2 in MC3T3-E1 cell line. **A** The sequences of miR-30a-5p binding site within the 3’UTR of Runx2 mRNA predicted by TargetScan, and the constructs of pmir-GLO-Runx2-WT luciferase reporter and pmir-GLO-Runx2-MUT control. **B** The relative luciferase activity in 293T cells after 24 h co-transfected with miR-30a-5p mimic (miR-30a-5p) or miR-NC and WT or MUT luciferase reporter was determined by dual-luciferase reporter assays. **C** qRT-PCR measurement of the Runx2 mRNA expressions after 24 h in MC3T3-E1 cells transfected with miR-30a-5p mimic (mimic) or miR-NC and miR-30a-5p inhibitor (inhibitor) or inhibitor NC. **D**, **E** RIP assay confirmed the binding between Runx2 and miR-30a-5p in MC3T3-E1 cell line. **F** RIP assay confirmed that there exists no binding between ALP miR-30a-5p in MC3T3-E1 cell line. **G** RIP assay confirmed that there exists no binding between OC between miR-30a-5p in MC3T3-E1 cell line. **H** RIP assay confirmed that there exists no binding between Col1 between miR-30a-5p in MC3T3-E1 cell line. All data are shown as mean ± SEM. *p < 0.05; **p < 0.01

The dual-luciferase report gene experiment is a direct method to verify whether the target genes are the target of miRNAs. The binding sites of miR-30a-5p and Runx2 were shown in Fig. 4A, and the dual-luciferase report gene results showed that Runx2 is a target of miR-30a-5p (Fig. 4B). The results showed that Runx2 increased in the mRNA level when transfecting miR-30a-5p mimics and declined with miR-30a-5p inhibitors (Fig. 4C). RIP experiment showed that miR-30a-5p and Runx2 assembled into Argonaute-2 protein, IgG was a negative control and input was a positive control (Fig. 4D, E), while miR-30-5p couldn't interact with ALP, OC, Col1 and then not assembled into Argonaute-2 protein (Fig. 4F–H). The results indicated that the action of miR-30a-5p in reducing the expression of ALP, OC and Col1 genes were indirect.

### Co-transfecting si-Runx2 and miR-30a-5p inhibitor combatted the effects of osteogenesis and inflammatory of si-Runx2 in MC3T3-E1 cell line by rescue experiments

**Fig. 5:**
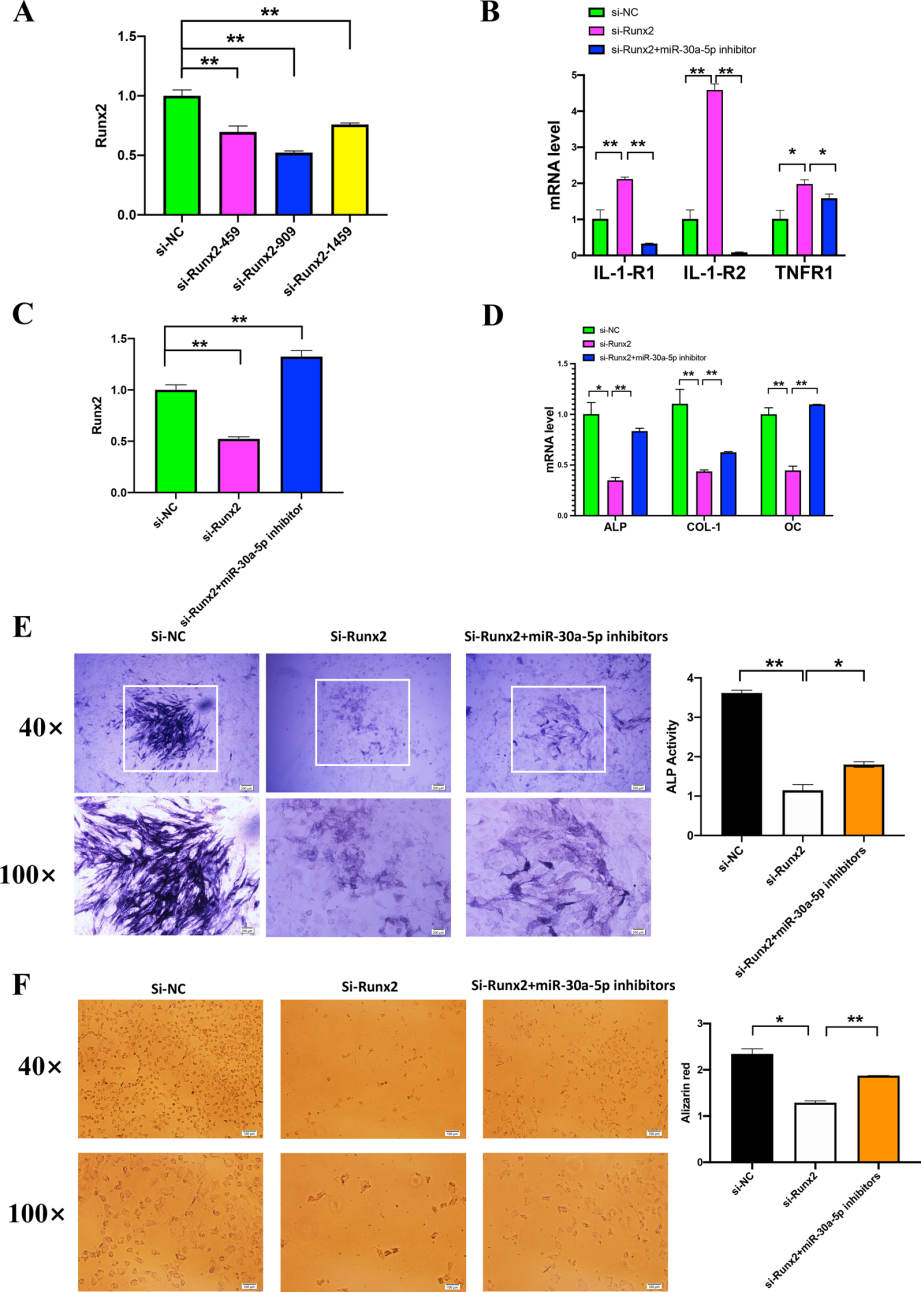
si-Runx2 plus miR-30a-5p inhibitors combatted the efficiency of osteogenesis and inflammation from si-Runx2. **A** The expression of Runx2 was examined by qPCR in MC3T3-E1 cells after 24 h after transfected with three si-Runx2 (si-Runx2-459, si-Runx2-909, si-Runx2-1459) and relative controls (si-NC). **B** The expression of Runx2 was examined by qPCR in MC3T3-E1 cells after 24 h after transfected with si-Runx2, relative controls (si-NC) and si-Runx2 + miR-30a inhibitors. **C** The expression of ALP, COL-1 and OC were examined by qPCR in MC3T3-E1 cells after 24 h after transfected with si-Runx2, relative controls (si-NC) and si-Runx2 + miR-30a inhibitors. **D** The expression of IL-1-R1, IL1-R2 and TNFR were examined by qPCR in MC3T3-E1 cells after 24 h after transfected with si-Runx2, relative controls (si-NC) and si-Runx2 + miR-30a inhibitors. **E** The level of ALP was examined by ALP staining in MC3T3-E1 cells after 7 days after transfected with si-Runx2, relative controls (si-NC) and si-Runx2 + miR-30a inhibitors. **F** The level of Alizarin red was examined by Alizarin red staining in MC3T3-E1 cells after 21 days after transfected with si-Runx2, relative controls (si-NC) and si-Runx2 + miR-30a inhibitors. All data are shown as mean ± SEM. *p < 0.05; **p < 0.01

qRT-PCR result showed that si-Runx2-909 silenced the expression of Runx2 efficiently (Fig. 5A), so it was utilized for following experiments and named si-Runx2. Inflammatory factors IL1R-1, IL1R-2 and TNFR upregulated when transfecting si-Runx2, however si-Runx2 plus miR-30a-5p inhibitors combatted the promoted effect (Fig. 5B). Osteogenesis factors Runx2, ALP, OC, COL1 (Fig. 5C, D) were downregulated when transfecting si-Runx2, however si-Runx2 plus miR-30a-5p inhibitors combatted inhibitory effect. Result of Alkaline phosphatase staining and alizarin red also indicated si-Runx2 plus miR-30a-5p inhibitor combatted the inhibitory osteogenesis effect of si-Runx2 (Fig. 5E, F).

## Discussion

Periodontitis, one of the multifactorial infectious diseases, is characterized by gingival inflammation and bone loss [14, 26]. Modern molecular technologies including pathogenic agents, haplotype aberrations and gene therapy have been used friendly in recent years [1]. For instance, CXCL12 (the chemokine stromal-cell derived factor-1) was increased in human periodontitis by targeting CXCR4. So, seeking a gene for inhibit inflammation and relieve bone loss makes it senses.

MiRNAs are evolutionarily conserved non-coding RNA molecules (20–22 nucleotides in length) that primarily function to prevent mRNA translation or initiate mRNA degradation at the posttranscriptional level via binding to the 3’ untranslated regions (3’ UTR) of their target mRNAs [27]. Besides, some animal miRNAs may also target 5’UTR and coding regions according to bioinformatics predictions and others experiments [28]. Mounting evidence showed that miRNAs involved in periodontal diseases including periodontitis recently [14, 29]. Lee et al. found six miRNAs were upregulated not only in inflammation but also in bone loss and injured early in 2001. miR-30a-5p, one of six miRNAs, which located in Chromosome 1: 23,311,350–23,311,420, was identified an amplified expression in periodontitis than healthy ones [13]. Furthermore, miR-30a-5p was a prognostic biomarker in colorectal cancer [30], a suppressor gene of non-small cell lung cancer [31]. miR-30a-5p was found to be relationship with allergic disease [32]. A recent study showed that miR-30a-5p, the mature product of miR-30a, promoted to exacerbate inflammation and organ damage in sepsis model [33].

In this study, miR-30a-5p was found to have an increasing expression in gingiva and bone tissues of the periodontitis rat model, comparing with that of normal periodontal tissue in vivo. In vitro, miR-30a-5p was equivalent upregulated in MC3T3-E1 cell line as time extension after LPS-inducing and reach at a higher level in 14 days testing by qRT-PCR. MiR-30a-5p were upregulated not only in soft tissue but also in hard tissue of periodontitis. All suggest that miR-30a-5p involved in process of periodontitis.

miR-30a-5p mimics, inhibitors were designed to transfect MC3T3 cell line. miR-30a-5p inhibitors promoted expression of the osteogenesis factors Runx2, ALP, OC, COL1 and reduced expression of inflammation factors IL1R-1, IL1R-2, TNFR, accompany with miR-30a-5p mimics played reversed roles, which suggested miR-30a-5p played a regulatory role in the osteogenesis and inflammation of periodontitis.

miRNAs modulate gene expression at the post-transcriptional level either by promoting mRNA degradation or by inhibiting messenger RNA (mRNA) translation [34]. According to the Target Scan websites (http://www.targetscan.org/mamm_31/), we found more than 1576 target genes of miR-30a-5p including Runx2 partly because it has a considerably long 3’-UTR (~ 4 kb). Runx2 is not only an inducer of chondrocyte and osteoblast differentiation but also a gene of mesenchymal precursor cell differentiation [21, 35]. The expression of Runx2 is distinct in different kinds of cells. For instance, Runx2 weakly expressed in uncommitted mesenchymal cells, highly in pre-osteoblasts, reached the maximal level in immature osteoblasts, and then down-regulated in mature osteoblasts [36]. Overexpression of Runx2 accelerated osteoblast differentiation under the regulation of the Prrx1 promoter [37, 38]. Additionally, microRNA-218 decreased the expression of inflammatory cytokines in acute lung injury model targeting Runx2 recent reports by Zhou et al. [32]. Therefore, Runx2 might be related to the inflammation. That is a potential reason for miR-30a-5p inhibitors promoting osteogenesis but inhibiting inflammation through the overexpression of Runx2. MiR-30a-5p alleviated the LPS-inducing inflammation by targeting Runx2 in A549 cell line [39]. Evidence whether miR-30a-5p plays a role in osteogenesis and inflammation by targeting Runx2 in MC3TC-E1 cell line need to be further explored.

We confirmed that Runx2 was a target gene of miR-30a-5p with certain binding sites by the dual luciferase gene reporter experiments. RIP is a direct method to detect the relationship between two different genes [40]. In this study, the expression of Argonaute-2 protein which directly link miR-30a-5p and Runx2 increased after transfecting miR-30a-5p mimics by RIP. RIP experiments also indicated that these are not direct conjunction between miR-30a-5p and ALP, OC, COL1. As shown in Fig. 5D ALP, OC and Col1 were up-regulated when transfecting with miR-30a-5p inhibitors because these genes were up-regulated with upregulation of Runx2. The mRNA expression level of Runx2 were downregulated with miR-30a-5p mimics, while upregulated with miR-30a-5p inhibitors. We also found that si-Runx2 inhibited osteogenesis and promoted inflammation, and miR-30a-5p inhibitor plus si-Runx2 could combat the inhibitory osteogenic differentiation and promoting inflammation of si-Runx2.

We must admit that lack of clinical data is a limitation for our research. And we have also cited a paper which pointed out that miR-30a-5p have higher expression level in periodontitis patients by microarray assay [13]. More detail validation methods need to be carried out in follow-up experiments. All dates in our study suggested that miR-30a-5p inhibitor would promote osteogenesis and relieve inflammation by targeting Runx2.

## Conclusions

**Fig. 6:**
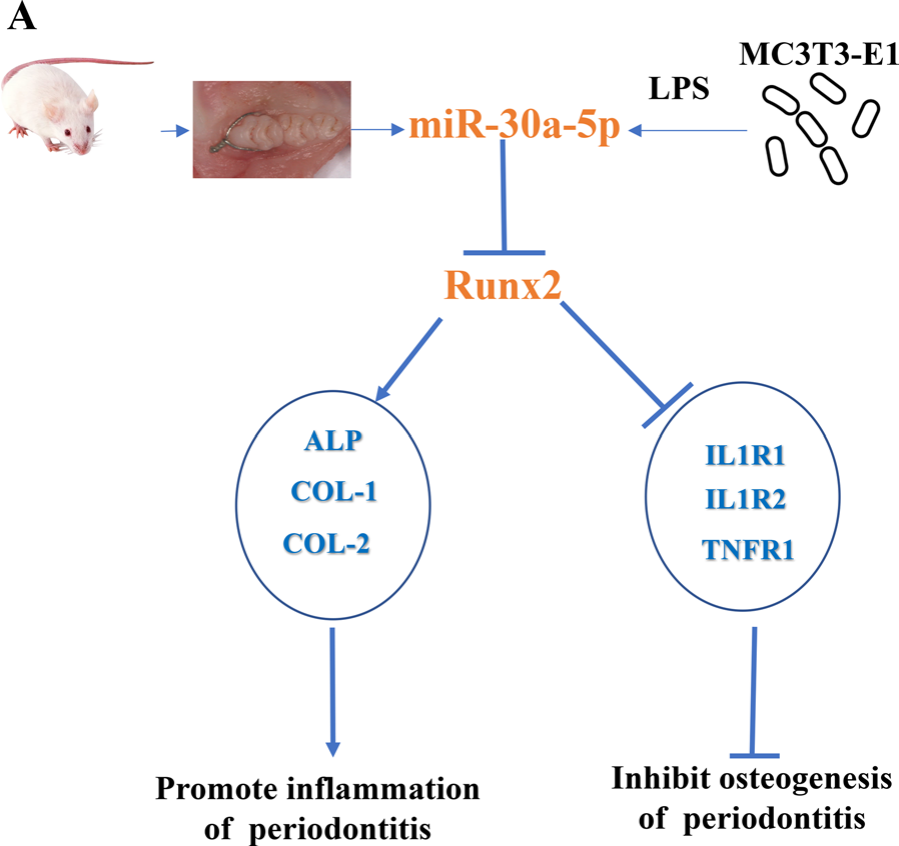
A diagram of the osteogenesis and inflammation functions of miR-30a-5p inhibited osteogenesis and promoted inflammation by targeting Runx2

In conclusion, miR-30a-5p was upregulated in inflammatory tissue of periodontitis rat model and MC3T3-E1 cell line, miR-30a-5p inhibitorS would promote osteogenesis and relieve inflammation by targeting Runx2 in MC3T3-E1 cell line under LPS-induing, which were summarized in Fig. 6. All suggested that miR-30a-5p involved in regulatory role of osteogenesis and inflammation targeting by Runx2 in periodontitis. Our study offered a potential means for promoting bone formation and provided a novel understanding of mechanism of periodontitis.

## Supplementary Information

**Additional file 1**. **Figure S1**. Runx2-WT-miR-30a-5p and Runx2-MUT-miR-30a-5p were cloned successfully. (**A**) A diagram of vector of GP-miRGLO for luciferase reporter gene experiment. (**B**) Sequencing of Runx2-WT-miR-30a-5p and Runx2-MUT-miR-30a-5p.

## Data Availability

The datasets used and/or analyzed during the current study are available from the corresponding author on reasonable request.

## Abbreviation

miRNA: MicroRNA
