# Supplementary material for: miR-30a-5p inhibits osteogenesis and promotes periodontitis by targeting Runx2
Source: BMC Oral Health. 2021 Oct 11;21:513. doi: 10.1186/s12903-021-01882-9 (PMC8504121; doi:10.1186/s12903-021-01882-9)
Supplement: Supplementary file 1 — Additional file 1. Figure S1. Runx2-WT-miR-30a-5p and Runx2-MUT-miR-30a-5p were cloned successfully. (A) A diagram of vector of GP-miRGLO for luciferase reporter gene experiment. (B) Sequencing of Runx2-WT-miR-30a-5p and Runx2-MUT-miR-30a-5p. [file 12903_2021_1882_MOESM1_ESM.pdf]

S1

A

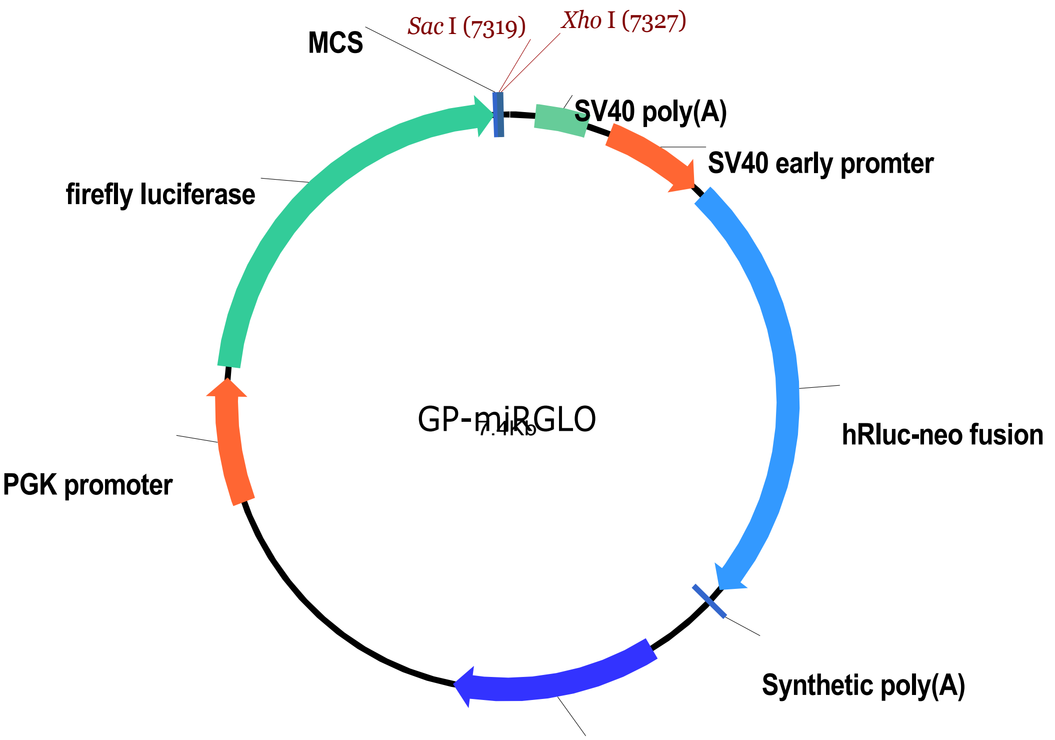

B

RUNX2-WT-miR-30a-5p

The most stable 3'-dimer: 62 bp, -103.9 kcal/mol

```
5' CGTGAGAATTTTTTAGATGTGTGTTTACTTCATGTTTACAAATAACTGTTTGCTTTTTAATC 3'
3' TCGAGCACTCTTAAAAAATCTACACACAAATGAAGTACAAATGTTTATTGACAAACGAAAAATTAGAGCT 5'
```

RUNX2-MUT-miR-30a-5p

The most stable 3'-dimer: 62 bp, -103.9 kcal/mol

```
5' CGTGAGAATTTTTTAGATGTGTGTTTACTTCAACAAATGTAATAACTGTTTGCTTTTTAATC 3'
3' TCGAGCACTCTTAAAAAATCTACACACAAATGAAGTTGTTTACATTATTGACAAACGAAAAATTAGAGCT 5'
```
